# Incidence of syphilis seroconversion among HIV-infected persons in Asia: results from the TREAT Asia HIV Observational Database

**DOI:** 10.7448/IAS.19.1.20965

**Published:** 2016-10-21

**Authors:** Jin Young Ahn, David Boettiger, Sasisopin Kiertiburanakul, Tuti Parwati Merati, Bui Vu Huy, Wing Wai Wong, Rossana Ditangco, Man Po Lee, Shinichi Oka, Nicolas Durier, Jun Yong Choi, on behalf of the Treat Asia HIV Observational Database

**Affiliations:** 1 Department of Internal Medicine, Yonsei University College of Medicine, Seoul, Korea; 2 AIDS Research Institute, Yonsei University College of Medicine, Seoul, Korea; 3 Department of Internal Medicine, Seoul Medical Center, Seoul, Korea; 4 The Kirby Institute, UNSW Australia, Sydney, Australia; 5 Faculty of Medicine, Ramathibodi Hospital, Mahidol University, Bangkok, Thailand; 6 Faculty of Medicine, Udayana University and Sanglah Hospital, Bali, Indonesia; 7 National Hospital for Tropical Diseases, Hanoi, Vietnam; 8 Taipei Veterans General Hospital, Taipei, Taiwan; 9 Research Institute for Tropical Medicine, Manila, Philippines; 10 Queen Elizabeth Hospital, Hong Kong, China; 11 National Center for Global Health and Medicine, Tokyo, Japan; 12 TREAT Asia, Foundation for AIDS Research, Bangkok, Thailand

**Keywords:** syphilis, incidence, seroconversion, HIV, MSM

## Abstract

**Introduction:**

Outbreaks of syphilis have been described among HIV-infected men who have sex with men (MSM) in Western communities, whereas reports in Asian countries are limited. We aimed to characterize the incidence and temporal trends of syphilis among HIV-infected MSM compared with HIV-infected non-MSM in Asian countries.

**Methods:**

Patients enrolled in the TREAT Asia HIV Observational Database cohort and with a negative non-treponemal test since enrolment were analyzed. Incidence of syphilis seroconversion, defined as a positive non-treponemal test after previously testing negative, was evaluated among patients at sites performing non-treponemal tests at least annually. Factors associated with syphilis seroconversion were investigated at sites doing non-treponemal testing in all new patients and subsequently testing routinely or when patients were suspected of having syphilis.

**Results:**

We included 1010 patients from five sites that performed non-treponemal tests in all new patients; those included had negative non-treponemal test results during enrolment and subsequent follow-ups. Among them, 657 patients were from three sites conducting regular non-treponemal testing. The incidence of syphilis seroconversion was 5.38/100 person-years (PY). Incidence was higher in MSM than non-MSM (7.64/100 PY vs. 2.44/100 PY, *p<*0.001). Among MSM, the incidence rate ratio (IRR) for every additional year from 2009 was 1.19 (*p=*0.051). MSM status (IRR 3.48, 95% confidence interval (CI) 1.88–6.47), past syphilis diagnosis (IRR 5.15, 95% CI 3.69–7.17) and younger age (IRR 0.84 for every additional 10 years, 95% CI 0.706–0.997) were significantly associated with syphilis seroconversion.

**Conclusions:**

We observed a higher incidence of syphilis seroconversion among HIV-infected MSM and a trend to increasing annual incidence. Regular screening for syphilis and targeted interventions to limit transmission are needed in this population.

## Introduction

After the development of penicillin, the number of syphilis cases fell to its lowest level in 2000 in the United States, from 20.3 cases per 100,000 people to 2.9 cases per 100,000 people. However, the overall number of new infection cases gradually increased after the early 2000s to 6.3 cases per 100,000 people in 2014 [1]. The World Health Organization reported that there were 10.6 million global new syphilis cases in 2008 [2]. Syphilis is also considered to be an significant problem in other specific areas, as more than a quarter of new cases of syphilis and other sexually transmitted infections have occurred in the Western Pacific region, including South Korea, Japan, Taiwan, China, Thailand, the Philippines and Australia [2]. Patients were predominantly male, and one surveillance reported that more than 60% of male patients were men who have sex with men (MSM) [1].

The estimated HIV prevalence in Asia is low (0.1% in East Asia and 0.3% in Southeast Asia) compared with other regions such as North America (0.5%) and Africa (4.7%) [3]. However, unlike the global decreasing trend of HIV prevalence, the estimated number of new cases has increased in East Asia since 2001 [3]. With regard to the transmission route, intravenous drug use, contaminated blood products and heterosexual contact initially played an important role in HIV transmission in Asian countries. However, the epidemic has recently changed, and the transmission of HIV among MSM has become a major threat in many Asian countries [4].

HIV and *Treponema pallidum* share similar routes of transmission. Syphilitic ulcers are known to disrupt the mucosal barriers, facilitating the passage of HIV [5, 6]. Syphilis is also associated with a decrease in CD4+ T lymphocyte and an increase in HIV viral load in co-infected patients [7, 8]. Some reports suggest that atypical or severe forms of syphilis are more frequent, and the course of syphilis more rapid, in HIV-infected patients [9–11].

In the 1990s, the incidence of syphilis in HIV-infected patients fell significantly, with enhanced screening, education and behavioural changes [12, 13]. However, a re-emergence of syphilis was reported in the 2000s in industrialized countries, especially among MSM [14–18]. In Western settings, a syphilis incidence of 2.9 to 6.2 per 100 PY has been reported in HIV-infected MSM [19–22]. The reasons for this resurgence of syphilis are complex, involving changes in risk behaviour and awareness of the need for testing [14, 18, 23–25]. Recently, the morbidity and mortality of HIV-infected patients has significantly decreased with prolonged life expectancy. This has enabled an increase in sexual activities, including risky sexual behaviours and serosorting of sexual partners among MSM [16, 26, 27]. Previous studies have reported that 33 to 52% of syphilis infections can be asymptomatic, creating additional challenges for diagnosis and stopping transmission [19, 28, 29]. To enhance identification and facilitate treatment in the context of concomitant HIV, US and European guidelines for management of HIV infection recommend routine screening of syphilis at least yearly among MSM [30–32].

However, routine screening of syphilis is limited in Asia. Many countries in the region have limited healthcare resources and the high false-positive rate of non-treponemal testing used for syphilis screening may make this approach unsuitable in this setting [33, 34]. Furthermore, data about rates of syphilis in Asian are inconsistent. There have been several reports of increasing syphilis co-infection among HIV-infected patients in Asian countries [4, 35, 36], while another study in Thailand found that only 1.7% of patients screened routinely had syphilis, suggesting the possible reduced importance of routine screening in this region [33]. As data remain limited, we aimed to determine trends in incidence and predictors of syphilis seroconversion, particularly among MSM compared to non-MSM, in a regional cohort of HIV-infected patients in Asia.

## Methods

### Study sites and population

The TREAT Asia HIV Observational Database (TAHOD) is a prospective, observational cohort study of HIV-infected patients enrolled from 21 clinical sites in 12 countries in the Asia–Pacific region, the details of which have been previously described [37]. Briefly, each site enrols 100 to 450 HIV-infected patients, both treated and untreated with antiretroviral therapy (ART). Data are collected according to a common protocol. On recruitment, all available retrospective data prior to enrolment in TAHOD are collected. Prospective data are biannually transferred to a central data management and biostatistical analysis centre. Institutional review board approvals are obtained at all participating sites, the data management and analysis centre (Kirby Institute, University of New South Wales, Sydney, Australia), and the coordinating centre (TREAT Asia/amfAR, Bangkok, Thailand). Patients provide written informed consent to participate in TAHOD, where required by local institutional review boards (IRBs).

Among the participating sites, five (one each in South Korea, Taiwan, Hong Kong, Japan and the Philippines) conduct syphilis testing of all new patients. All five countries have only one contributing site each. Three of these sites (in South Korea, Taiwan and Hong Kong) perform routine testing for syphilis at least yearly, whereas the other two sites perform testing only when patients are symptomatic or suspected of having syphilis (Table 1). We investigated the incidence of syphilis seroconversion at sites that test patients for syphilis at least annually (*n*=3) and described factors associated with syphilis seroconversion at sites that test all new patients (*n*=5). Patients with a negative Venereal Disease Research Laboratory (VDRL) test or rapid plasma regain (RPR) test after enrolment into TAHOD were eligible for inclusion in the analysis.

**Table 1:** Syphilis testing practices at participating study sites

Site country	Number of contributing patients	Testing new patients	Testing symptomatic or suspected patients	Regular screening	Increased test frequency for any patients?	Median number of reported tests per patient per year (IQR)
South Korea	177	Yes	Yes	Every six months	No	2.07 (1.55–2.65)
Taiwan	343	Yes	Yes	Minimum every 12 months	Every six months for those subjectively assessed to be at high risk	1.17 (0.07–2.18)
Hong Kong	137	Yes	Yes	Minimum every 12 months	Every six months for MSM	1.84 (0.15–2.80)
Japan	83	Yes	Yes	No	No	0.54 (0.24–1.13)
Philippines	270	Yes	Yes	No	No	0.62 (0.40–0.84)

IQR, interquartile range; MSM, men who have sex with men.

### Study variables and definitions

The study end point was syphilis seroconversion, defined as a positive VDRL or RPR test after previously testing negative during TAHOD enrolment. VDRL and RPR tests appear to be accurate and reliable for diagnosis and monitoring treatment response in most HIV-infected patients [38]. The sensitivity of the non-treponemal test is known to be 78 to 86% in primary syphilis and 100% in secondary syphilis, with 98% specificity [38, 39]. Either VDRL or RPR tests were performed on patients according to the testing policy of each site. The positive VDRL and PRP tests included the results of both qualitative and quantitative tests. Study variables included age, sex, ethnicity, mode of HIV exposure, hepatitis B and C serology, history of prior AIDS diagnosis, CD4 cell count, HIV viral load, highly active antiretroviral therapy (HAART) regimen and past history of having positive VDRL or RPR test prior to baseline. Variables were measured at baseline. Baseline was considered as the date of first negative VDRL or RPR test during TAHOD enrolment.

### Statistical analysis

Patients were only censored at the first positive syphilis test and not able to contribute more than one outcome. Follow-up was censored at the last available clinic visit date without a record of seroconversion. The total and annual incidence of syphilis seroconversion was evaluated overall and by MSM status (MSM/non-MSM). Trends in syphilis incidence were evaluated by univariate Poisson regression. Predictors of syphilis seroconversion were evaluated by Poisson regression adjusted for study site. In the analysis of predictors of syphilis seroconversion, covariates significant in the univariate model at *p*<0.10 were chosen for inclusion in the multivariate model. Covariates with *p<*0.05 in the final multivariate model were considered statistically significant. All models were adjusted for each study site, although the incidence rate ratio (IRR) for each site is not shown. Missing categories were included in the models; however, the IRRs are not reported. Stata statistical software (version 12.1; StataCorp, College Station, TX, USA) was used for all statistical analyses.

## Results

From September 2003 to March 2014, data from 2135 patients receiving HIV care at one of the five sites were available for this analysis. Overall, 1047 (49.0%) patients had a negative VDRL or RPR test during enrolment, and 1010 (96.4%) had subsequent follow-up testing data. Among the three sites that routinely tested for syphilis, 1359 patients were enrolled in TAHOD, 691 (50.8%) had a negative VDRL or RPR result during enrolment and 657 (95.1%) had subsequent follow-up data. The demographics and characteristics of all patients (*n*=1010) and those considered for the incidence analysis (*n*=657) are shown in Table 2. Both groups were predominantly male. Median age, proportions having prior AIDS and using HAART at the time of syphilis seroconversion were similar among both groups. Homosexual contact was the most common mode of HIV exposure (63.0% in all patients and 65.4% in the incidence group). The median baseline CD4 cell count was 393 cells/mm^3^ (interquartile range (IQR) 253–551) among the overall group of patients and 410 cells/mm^3^ (IQR 259–561) in the incidence group. Twenty percent of the overall group and 24.7% of the incidence group had a history of syphilis infection before enrolment.

**Table 2:** Baseline characteristics

Factors	Total patients (*n*=1010)	Incidence subgroup (*n*=657)
Male, *n* (%)	933 (92.4)	621 (94.5)
Age (years), *n* (%)		
≤30	235 (23.3)	138 (21.0)
31–40	335 (33.2)	223 (33.9)
41–50	276 (27.3)	181 (27.5)
>50	164 (16.2)	115 (17.5)
Median (IQR)	38.2 (30.3–45.6)	38.5 (31.1–45.7)
Ethnicity, *n* (%)		
Asian	1003 (99.3)	653 (99.4)
Caucasian	5 (0.5)	4 (0.6)
Other	2 (0.2)	0 (0.0)
HIV exposure, *n* (%)		
Heterosexual	230 (22.8)	136 (20.7)
MSM	636 (63.0)	430 (65.4)
Injecting drug use	11 (1.1)	10 (1.5)
Other	133 (13.2)	81 (12.3)
Prior AIDS diagnosis, *n* (%)	354 (35.0)	235 (35.8)
CD4 cell count (cells/mm^3^), *n* (%)		
≥500	301 (29.8)	215 (32.7)
350–499	248 (24.6)	171 (26.0)
200–349	236 (23.4)	152 (23.1)
<200	154 (15.2)	95 (14.5)
Median (IQR)	393 (253–551)	410 (259–561)
HIV viral load (copies/ml), *n* (%)		
<400	546 (54.1)	439 (66.8)
≥400	258 (25.5)	171 (26.0)
Median (IQR)	49 (39–3410)	49 (39–950)
Median years after HIV diagnosis (IQR)	3.59 (1.5–6.6)	3.54 (1.6–6.6)
Using HAART at baseline, *n* (%)	887 (87.8)	570 (86.8)
Positive HBsAg, *n* (%)	101 (11.9)	70 (13.4)
Positive hepatitis C antibody, *n* (%)	43 (5.4)	34 (6.0)
Past syphilis diagnosis, *n* (%)	202 (20.0)	162 (24.7)

IQR, interquartile range; MSM, men who have sex with men; HAART, highly active antiretroviral therapy; HBsAg, hepatitis B surface antigen.

### Incidence analysis

During the study period, there were a total of 127 cases of syphilis seroconversion in the incidence group. The total follow-up duration was 2359 person-years (PY) and the median follow-up time per patient was 2.92 (IQR 1.08–5.69) years. A median of 1.59 (IQR 0.80–2.54) VDRL or RPR tests per patient per year were performed. The overall incidence of syphilis seroconversion was 5.38 (95% confidence interval (CI) 4.52–6.41) per 100 PY. When assessed by MSM status, the incidence of seroconversion was 2.44 (95% CI 1.65–3.61) per 100 PY among non-MSM and 7.64 (95% CI 6.29–9.28) per 100 PY among MSM (*p<*0.001).

**Figure 1:**
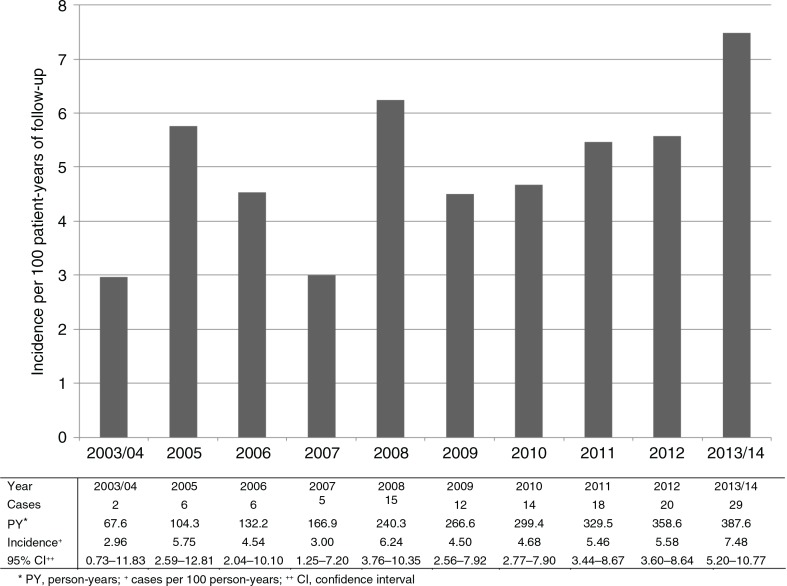
Incidence of syphilis seroconversion among HIV-infected patients by year (*n*=657).

**Figure 2:**
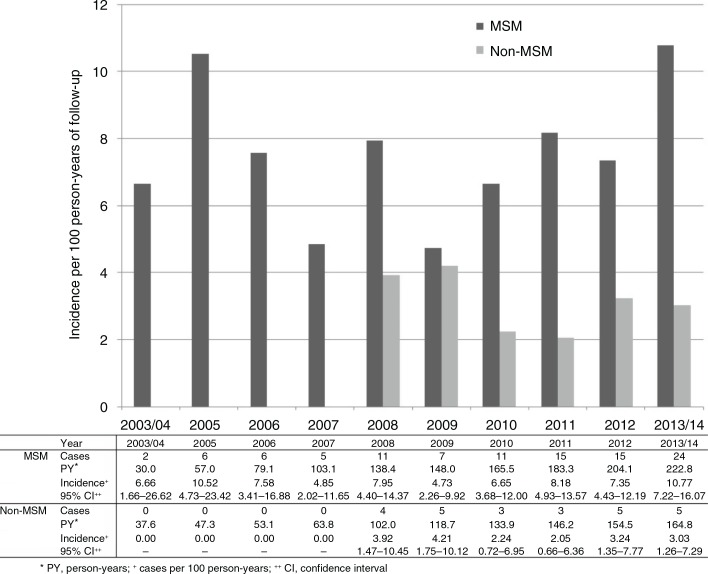
Incidence of syphilis seroconversion among MSM and non-MSM by year (*n*=657). MSM, men who have sex with men.

Over the study period, the rate of syphilis seroconversion was lowest in 2003 to 2004 at 2.96/100 PY and tended to show an increasing trend over time (Figure 1). Incidence was 7.48/100 PY in the period from 2013 to 2014. In particular, Figure 1 indicates a recent increase from 2009 with a visually ascending trend in annual incidence. From 2009 onwards, the univariate IRR for every additional year was 1.14 (95% CI 0.98–1.32, *p=*0.091). Yearly change in incidence was also evaluated by MSM status (Figure 2). In any given year, incidence was consistently higher among the MSM patients. In this group, incidence reached a peak value of 10.77/100 PY in 2013 to 2014. The univariate IRR for every additional year from 2009 onwards was 1.19 in MSM (95% CI 1.00–1.41, *p=*0.051) and 0.97 (95% CI 0.71–1.31, *p=*0.838) in non-MSM.

### Factors associated with syphilis seroconversion

Factors associated with syphilis seroconversion among all patients are shown in Table 3. In the multivariate analysis, HIV exposure via MSM contact (IRR 3.48 vs. heterosexual, 95% CI 1.88–6.47, *p<*0.001), past diagnosis of syphilis (IRR 5.15 vs. none, 95% CI 3.69–7.17, *p<*0.001) and younger age (IRR 0.84 for every additional 10 years, 95% CI 0.706–0.997, *p*=0.047) were significantly associated with syphilis seroconversion.

**Table 3:** Factors associated with syphilis seroconversion among HIV-infected patients (*n*=1010)

Baseline risk factor	Univariate IRR (95% CI)	*p*	Multivariate IRR (95% CI)	*p*
HIV exposure				
Heterosexual	1		1	
MSM	5.50 (2.96–10.22)	<0.001	3.48 (1.88–6.47)	<0.001
Injecting drug use	1.61 (0.21–12.52)	0.649		
Other	3.39 (1.57–7.34)	0.002	2.50 (1.16–5.42)	0.02
Past syphilis diagnosis				
No	1		1	
Yes	6.06 (4.37–8.39)	<0.001	5.15 (3.69–7.17)	<0.001
Age, every 10-year increase	0.83 (0.70–0.98)	0.029	0.84 (0.71–1.00)	0.047
Sex				
Male	1			
Female	0.07 (0.01–0.54)	0.01		
Prior AIDS diagnosis				
Not known	1			
Yes	0.76 (0.54–1.09)	0.134		
CD4 cell count (cells/mm3)				
≥500	1			
350–499	1.14 (0.75–1.73)	0.549		
200–349	0.94 (0.61–1.44)	0.775		
<200	1.19 (0.71–1.98)	0.515		
HIV viral load (copies/ml)				
<400	1			
≥400	0.94 (0.65–1.36)	0.742		
Time after HIV diagnosis				
Every one-year increase	1.02 (0.98–1.06)	0.295		
Using HAART				
Yes	1			
No	0.88 (0.56–1.39)	0.593		
HBsAg status				
Negative	1			
Positive	1.34 (0.84–2.14)	0.223		
Hepatitis C antibody status				
Negative	1			
Positive	0.95 (0.44–2.05)	0.888		

CI, confidence interval; IRR, incidence rate ratio; MSM, men who have sex with men; HAART, highly active antiretroviral therapy; HBsAg, hepatitis B surface antigen. All models were adjusted for study site, though incident rate ratios for sites are not shown.

## Discussion

Data about syphilis incidence from Asian countries have been limited and have shown inconsistent results [33, 35, 36, 40]. Most studies in Asia have only reported prevalence figures among HIV-infected patients or syphilis incidence in MSM regardless of HIV infection status. In a single centre study in Thailand, the prevalence of syphilis in HIV-infected patients was reported as 1.7% [33]. However, in that study, only 14.6% of the patients had reported homosexual contact risk, and the frequency of sexual contact may have further influenced the results (55.6% of the overall patients and 84% of MSM reported no recent sexual intercourse).

In our study, the overall incidence of syphilis seroconversion was higher than expected based on previous reports, which may be related to the higher proportion of MSM in our study population. Syphilis seroconversion was more frequent in the MSM group, and yearly incidence showed an increasing trend after 2009.

There are several possible reasons for the increases in syphilis seroconversions. Multiple sexual partners, use of drugs like methamphetamine or meeting partners over the Internet have been shown to increase risks of syphilis infection in HIV patients [41, 42]. Serial data suggests that risky behaviours are increasing in some HIV-infected MSM populations where syphilis rates are rising [26, 43, 44]. In one study from California, the proportion of HIV-infected MSM having 10 or more sexual partners or engaging in unprotected anal intercourse in the previous six months was reported to have increased in the early 2000s [45]. In addition, it has been shown that MSM can select their sexual partners or behaviours on the basis of their HIV infection status, in a practice known as serosorting [46–49]. This may be used to avoid the risk of transmitting HIV to an HIV-uninfected partner but could be associated with the risk of sexually transmitted infections including syphilis because risky sexual behaviour can be more common among HIV-infected MSM [46, 50–52].

In many countries, preventive interventions for HIV and syphilis among MSM have primarily targeted the HIV uninfected [46, 53]. However, the high incidence of syphilis among HIV-infected MSM in other contexts and observed in our cohort suggests that additional efforts are needed to support prevention within the MSM community.

In addition to reducing risky behaviours, another key component of a public health strategy to address syphilis in HIV-infected individuals is early detection and prompt treatment. Current US and European guidelines recommend at least annual screening for sexually transmitted infections, including syphilis, in sexually active HIV-infected patients [30, 31]. In our cohort, only 3 of 21 sites performed regular syphilis screening on all or even a subset of patients. Our findings indicate that regional health programmes may need to consider implementing routine screening in those with HIV.

There were several limitations to our study. Firstly, we considered that syphilis seroconversion defined as a newly positive VDRL or RPR test represented syphilis infection. Current guidelines recommend performing both treponemal tests and non-treponemal tests for syphilis diagnosis [31, 54]. Non-treponemal tests can have high rates of false positivity, which could overestimate the incidence of syphilis [55]. Secondly, the scope of data collection in our observational cohort did not allow for an assessment of clinical manifestations or of titres of non-treponemal tests; therefore, we could not establish whether there was a fourfold increase in the VDRL or RPR titre in order to define a syphilis infection among those with a history of syphilis and clinical diagnosis, such as primary or secondary syphilis. Similarly we were unable to correlate sexual behaviour information beyond the reported HIV exposure category or history of other sexually transmitted infections to syphilis seroconversions. In addition, only 5 of the 21 sites in our cohort conducted routine syphilis screening and were included in our analysis, and 4 of these were in high-income economies, preventing extrapolation to low- and middle-income settings.

Despite these limitations, this is the first multicentre study investigating syphilis infection among HIV-infected patients in Asia, where the epidemiology of HIV and syphilis and socio-economic status differ from Western settings. Understanding local disease epidemics and recent trends of incidence is essential for prevention and interventions.

## Conclusions

The high incidence of syphilis seroconversion observed in our study, especially among HIV-infected MSM, may represent high rates of transmissions and ongoing risky sexual behaviours, and it highlights the need for targeted intervention and regular syphilis screening in this population.

The TREAT Asia HIV Observational Database

CV Mean, V Saphonn* and K Vohith, National Center for HIV/AIDS, Dermatology and STDs, Phnom Penh, Cambodia; FJ Zhang*, HX Zhao and N Han, Beijing Ditan Hospital, Capital Medical University, Beijing, China; MP Lee*‡, PCK Li, W Lam, and YT Chan, Queen Elizabeth Hospital, Hong Kong, China; N Kumarasamy*, S Saghayam and C Ezhilarasi, Chennai Antiviral Research and Treatment Clinical Research Site, YRGCARE Medical Centre, VHS, Chennai, India; S Pujari*, K Joshi, S Gaikwad and A Chitalikar, Institute of Infectious Diseases, Pune, India; TP Merati*†, DN Wirawan and F Yuliana, Faculty of Medicine, Udayana University and Sanglah Hospital, Bali, Indonesia; E Yunihastuti*, D Imran and A Widhani, Working Group on AIDS, Faculty of Medicine, University of Indonesia/Cipto Mangunkusumo Hospital, Jakarta, Indonesia; S Oka*, J Tanuma and T Nishijima, National Center for Global Health and Medicine, Tokyo, Japan; JY Choi*, S Na and JM Kim, Division of Infectious Diseases, Department of Internal Medicine, Yonsei University College of Medicine, Seoul, South Korea; BLH Sim*, YM Gani and R David, Sungai Buloh Hospital, Sungai Buloh, Malaysia; A Kamarulzaman*, SF Syed Omar, S Ponnampalavanar and I Azwa, University of Malaya Medical Centre, Kuala Lumpur, Malaysia; R Ditangco*, E Uy and R Bantique, Research Institute for Tropical Medicine, Manila, Philippines; WW Wong*, WW Ku and PC Wu, Taipei Veterans General Hospital, Taipei, Taiwan; OT Ng*, PL Lim, LS Lee and PS Ohnmar, Tan Tock Seng Hospital, Singapore; P Phanuphak*, K Ruxrungtham, A Avihingsanon, P Chusut and S Sirivichayakul, HIV-NAT/Thai Red Cross AIDS Research Centre, Bangkok, Thailand; S Kiertiburanakul*, S Sungkanuparph, L Chumla and N Sanmeema, Faculty of Medicine, Ramathibodi Hospital, Mahidol University, Bangkok, Thailand; R Chaiwarith*, T Sirisanthana, W Kotarathititum and J Praparattanapan, Research Institute for Health Sciences, Chiang Mai University, Chiang Mai, Thailand; P Kantipong* and P Kambua, Chiangrai Prachanukroh Hospital, Chiang Rai, Thailand; W Ratanasuwan* and R Sriondee, Faculty of Medicine, Siriraj Hospital, Mahidol University, Bangkok, Thailand; VK Nguyen*, VH Bui, THD Nguyen and TD Nguyen, National Hospital for Tropical Diseases, Hanoi, Vietnam; TT Pham*, DD Cuong and HL Ha, Bach Mai Hospital, Hanoi, Vietnam; AH Sohn*, N Durier* and B Petersen, TREAT Asia, The Foundation for AIDS Research, Bangkok, Thailand; DA Cooper, MG Law*, A Jiamsakul* and DC Boettiger, The Kirby Institute, University of New South Wales (UNSW) Australia, Sydney, Australia.

*TAHOD steering committee member; †steering committee chair; ‡co-chair.
